# Resibufogenin Suppresses Triple-Negative Breast Cancer Angiogenesis by Blocking VEGFR2-Mediated Signaling Pathway

**DOI:** 10.3389/fphar.2021.682735

**Published:** 2021-04-30

**Authors:** Ting Yang, Yi-Xin Jiang, Ye Wu, Dong Lu, Rui Huang, Long-Ling Wang, Shi-Qi Wang, Ying-Yun Guan, Hong Zhang, Xin Luan

**Affiliations:** ^1^ Institute of Interdisciplinary Integrative Medicine Research, Shanghai University of Traditional Chinese Medicine, Shanghai, China; ^2^ Department of Pharmacy, Ruijin Hospital, School of Medicine, Shanghai Jiaotong University, Shanghai, China

**Keywords:** resibufogenin, antiangiogenic cancer therapy, VEGFR2, tumor angiogenesis, triple-negative breast cancer

## Abstract

Resibufogenin (RBF), an active compound from *Bufo bufonis*, has been used for the treatment of multiple malignant cancers, including pancreatic cancer, colorectal cancer, and breast cancer. However, whether RBF could exert its antitumor effect by inhibiting angiogenesis remains unknown. Here, we aimed to explore the antiangiogenic activity of RBF and its underlying mechanism on human umbilical vein endothelial cell (HUVEC), and the therapeutic efficacy with regard to antiangiogenesis *in vivo* using two triple-negative breast cancer (TNBC) models. Our results demonstrated that RBF can inhibit the proliferation, migration, and tube formation of HUVECs in a dose-dependent manner. Spheroid sprouts were thinner and shorter after RBF treatment *in vitro* 3D spheroid sprouting assay. RBF also significantly suppressed VEGF-mediated vascular network formation *in vivo* Matrigel plug assay. In addition, Western blot analysis was used to reveal that RBF inhibited the phosphorylation of VEGFR2 and its downstream protein kinases FAK and Src in endothelial cells (ECs). Molecular docking simulations showed that RBF affected the phosphorylation of VEGFR2 by competitively binding to the ATP-bound VEGFR2 kinase domain, thus preventing ATP from providing phosphate groups. Finally, we found that RBF exhibited promising antitumor effect through antiangiogenesis *in vivo* without obvious toxicity. The present study first revealed the high antiangiogenic activity and the underlying molecular basis of RBF, suggesting that RBF could be a potential antiangiogenic agent for angiogenesis-related diseases.

## Introduction

Updated Global Cancer Statistics indicated that female breast cancer has surpassed lung cancer as the leading cause of global cancer incidence in 2020 with an estimated 2.3 million new cases, representing 11.7% of all cancer cases (Sung et al., 2021). Triple-negative breast cancer (TNBC) is the most challenging heterogenous subtype of breast cancer often associated with an aggressive phenotype, high recurrence, metastasis, and poor prognosis (Bianchini et al., 2016). Approximately 12% of breast cancer patients are TNBC in the United States from 2012 to 2016, with a 5-year survival rate is 8–16% lower than other subtypes (DeSantis et al., 2019; Howard and Olopade, 2021). Owing to the absence of expression of the estrogen receptor (ER), progesterone receptor (PgR), and human epidermal growth factor receptor 2 (HER2), conventional cytotoxic chemotherapy remains the mainstay of treatment (Waks and Winer, 2019). However, chemotherapeutics may cause the acute nonspecific side effects for normal tissues and multidrug resistance (MDR), leading to therapeutic failure (Nedeljkovic and Damjanovic, 2019). Therefore, discovery of neoadjuvant drugs with highly selective antitumor mechanism has become a promising approach for the treatment of TNBC.

Angiogenesis plays a critical role in tumor formation, progression, and metastasis. Through excessive secretion of pro-angiogenic factors, tumor cells continue to activate ECs to “sprout” in the original blood vessels and form new vascular structures (Duran et al., 2017). Correspondingly, angiogenesis provides cancer cells with the essential nutrients and oxygen, and also a route for metastasis (Viallard and Larrivée, 2017). Therefore, inhibiting tumor angiogenesis keeps an attractive strategy for oncotherapy since decades ago. To date, bevacizumab is the only antiangiogenic drug approved by FDA (Food and Drug Administration) for TNBC (Xie et al., 2021), whereas bevacizumab has little effect on overall survival due to acquired drug resistance (Liu et al., 2020) and its limitation of blocking VEGFA expression (Zou et al., 2020). It is essential to find antiangiogenic medicines with novel skeleton for antiangiogenesis therapy and to overcome drug resistance.

**FIGURE 1:**
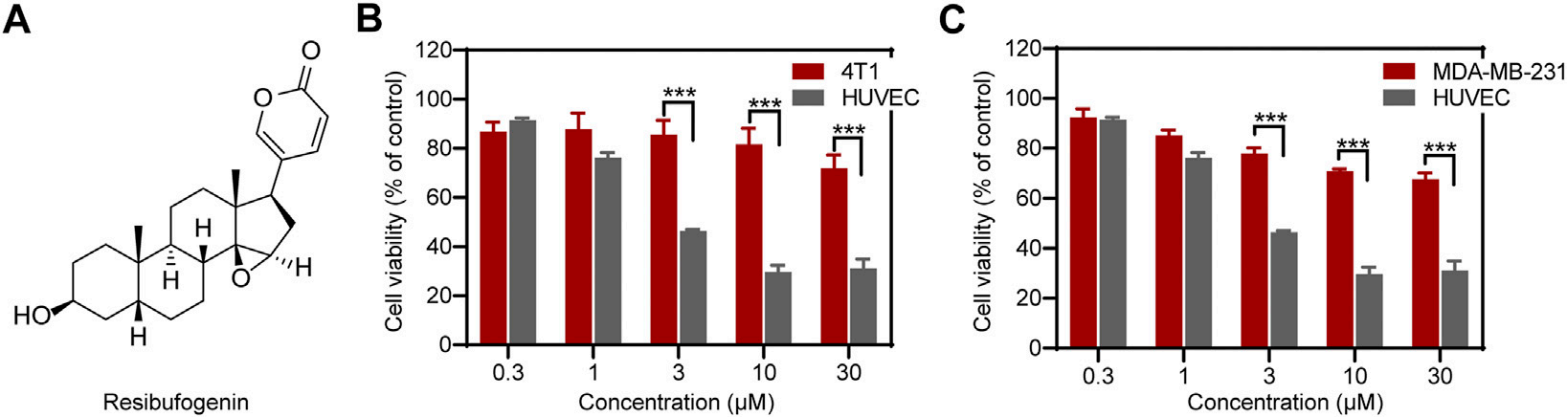
RBF effectively inhibited the viability of HUVECs relative to 4T1 and MDA-MB-231 cells. **(A)** The chemical structure of RBF drawn through ChemDraw 19.0. **(B, C)** RBF dose-dependently inhibited the viability of HUVECs at concentrations without affecting 4T1 and MDA-MB-231 cells. Values are expressed as mean ± SD, *n* = 4. ***p < 0.001 *vs.* 4T1 or MDA-MB-231 cells.

Natural products provide unparalleled source with unique molecular scaffolds for antiangiogenic drug discovery. Among them, resibufogenin (RBF) (Figure 1A), the main component of the antitumor traditional Chinese medicine (TCM) *Bufo bufonis* from the dry secretions of *Bufo gargarizans Cantor* and *Bufo melanostictus Schneider* (Chu et al., 2016), is a compound with the steroid mother nucleus structure of cardiotonic aglycone (Qi et al., 2011). Studies have shown that RBF has antitumor activity and can inhibit the tumor growth through different mechanisms. For instance, RBF can suppress transforming growth factor-β activated kinase 1 (Tak1)–mediated nuclear transcription factor-kappa B (NF-κB) activity through protein kinase C–dependent inhibition of glycogen synthase kinase-3 (GSK-3) on pancreatic cancer cells, PANC-1 and ASPC (Liu et al., 2018). RBF also can inhibit the growth and metastasis of colorectal cancer by triggering RIP3-dependent necrotizing ptosis and inducing glutathione peroxidase 4 (Gpx4) inactivation to induce oxidative stress (Han et al., 2018; Shen et al., 2021). Moreover, RBF treatment exhibited antitumorigenic and anti-Warburg effect in breast cancer through upregulating the inhibitory effect of miR-143–3p/HK2 axis (Guo et al., 2020). However, there is no study on RBF-mediated regulation of angiogenesis, the essential step for TNBC growth.

In present study, we first investigated the antiangiogenic effect of RBF and its mechanism on human umbilical vein endothelial cell (HUVEC). The antiangiogenic activity of RBF *in vivo* was evaluated by Matrigel plug assay. Furthermore, the *in vivo* antiangiogenic efficacy of RBF was evaluated in 4T1 and MDA-MB-231 orthotopic mice models. This study provided a new theoretical basis and reference for the potential clinical application of RBF.

## Materials and Methods

### Materials, Cell Lines, and Animals

RBF was purchased from Wuhan Zhongbiao Technology Company (Wuhan, China). Recombinant human vascular endothelial growth factor (VEGF165), Rabbit polyclonal antibodies against phospho-VEGFR-2, VEGFR-2, FAK, phosphor-FAK, Src, and phosphor-Src were obtained from Cell Signaling Technology (Danvers, MA).

Breast cancer cell lines 4T1 and MDA-MB-231 were obtained from Shanghai Cell Bank, Chinese Academy of Sciences (Shanghai, China). 4T1 cells were cultured in RPMI 1640 medium (meilunbio, Dalian, China), and MDA-MB-231 cells were cultured in Leibovitz’s L-15 medium (Gibco, United States). All culture media were supplemented with 10% fetal bovine serum (FBS, Gibco, United States), 1% penicillin, and 1% streptomycin. Human umbilical vein endothelial cells (HUVECs) were obtained using Lifeline Cell Technology and cultured in completed endothelial cell medium (Lifeline^®^ Cell Technology, Frederick, MD). 4T1 cells and HUVECs were cultured at 37°C in a humidified atmosphere containing 5% carbon dioxide (CO_2_), and MDA-MB-231 cells were cultured at 37°C without CO_2_.

BALB/c miceBALB/c nude mice, and C57 BL/6 mice were supplied by Shanghai Laboratory Animal Center (Shanghai, China). The animals were kept in an environment-controlled room (temperature: 20–25°C, relative humidity: 55–65%, and 12 h light/12 h dark cycle) with free access to water and fodder. All animal experimental protocols were approved by the Animal Ethics Committee of Shanghai University of Traditional Chinese Medicine.

### Cell Viability Assay

The effect of RBF on cell viability of the HUVECs, 4T1 and MDA-MB-231 cells was measured using Cell Counting Kit-8 (CCK-8, meilunbio, Dalian, China). Briefly, these cells were seeded in 96 well plates (5 × 10^3^ cells/well), respectively. After 24–h incubation, the cells were treated with different concentrations of RBF (0.3, 1, 3, 10, and 30 μM) for 24 h. Then, 100 μl medium containing 10% CCK-8 was added to each well and incubated at 37°C for additional 2 h. The absorbance at 450 nm was determined by microplate reader (Spark 10M, Tecan, Switzerland). The percentage of cell viability was calculated against control. Each condition included replicate wells with at least four independent repeats.

### Endothelial Cell Wound Healing Assay

HUVECs (2 × 10^5^ cells/well) were seeded in a 6-well plate and incubated at 37°C for 24 h. Subsequently, confluent HUVECs were scratched with the pipette tips, washed with PBS and photographed, and then the cells were treated with various concentrations of RBF (0.3, 1, 3, 10, and 30 μM). After drug stimulation for 12 h, the plate was photographed with microscope (Spark 10M, Tecan, Switzerland) and EC migration was quantified by Image-Pro Plus 6.0 software (Media Cybernetics, Bethesda, MD).

### Endothelial Cell Tube Formation Assay

Tube formation assay was carried out as described previously with some modifications (Dai et al., 2016; Lu et al., 2017). In brief, a precooled 96-well plate was coated with 50 μl/well Matrigel (BD Biosciences, San Jose, CA), which was thawed at 4°C overnight in advance and then incubated at 37°C for at least 30 min. HUVECs (1 × 10^4^ cells/well) were dispersed in the completed medium containing different concentrations of RBF (0.3, 1, 3, and 10 μM) and then seeded on the Matrigel layer. After 10 h of incubation (37°C with 5% CO_2_), the tubular structure formed by HUVECs stained with calcein AM with a final concentration of 2 μM for 15 min, then fluorescence photography was performed with Cytation 5 (BioTek, United States). The tube length was quantified by Image Pro Plus 6.0 software.

### Endothelial Cell Transwell Migration Assay

The chemotactic motility of HUVEC was investigated using a transwell migration assay with 24-well transwell plates of polycarbonate filter with 8 μm pore diameter and 6.5 mm diameter inserts. Briefly, complete medium containing 20 ng/ml VEGF165 was added to the lower chamber, and HUVECs (2 × 10^4^ cells/well) was suspended in the medium which containing different concentrations of RBF (0.3, 1, 3, and 10 μM) and seeded in the top chamber. After incubation for 8 h in an incubator (37°C with 5% CO_2_), the migrated cells were fixed with 4% paraformaldehyde for 20 min, then stained with 0.1% crystal violet, while the nonmigrated cells on the upper surface of polycarbonate membrane were gently wiped off with a cotton swab. The cells on the other side of the membrane were photographed under an inverted microscope (Laica, Germany) after washing the membrane three times with PBS. The number of migrated cells was determined by Image-Pro Plus 6.0 software.

### Spheroid-Based Angiogenesis Assay

ECs spheroids of defined cell number were generated as described previously (Heiss et al., 2015; Wu et al., 2019) with minor modifications. In brief, HUVECs (1.6 × 10^4^ cells/ml) were suspended in a culture medium containing 0.24% (wt/vol) methylcellulose (Adamas, China) and the mixture seeded alternately in a 100-mm × 20-mm dish (Corning, United States). Under these conditions, all suspended cells contributed to the formation of a single spheroid of defined size and cell number (400 cells/spheroid). Spheroids were cultured for 24 h in an incubator (37°C with 5% CO_2_). Afterward, the spheroids were suspended with a solution of rat tail collagen type I (BD, United States), then rapidly transferred into prewarmed 24-well plates and allowed to polymerize (30 min). After the collagen gels were set, 100 μl of complete medium containing 500 ng/ml VEGF_165_ and different concentrations of RBF (1 or 3 μM) or complete medium only containing 500 ng/ml VEGF165 was added to each well, and the spheroids formed sprouts after 24 h. The sprouts were photographed with microscope (Spark 10 M, Tecan, Switzerland).

### Matrigel Plug Assay

Six-week-old female C57BL/6 mice were subcutaneously injected with Matrigel mixture (400 μl/plug) containing 400 ng/ml VEGF and different concentrations of RBF (10 or 30 μM), and the Matrigel was mixed with PBS for mock control or 400 ng/ml VEGF for vehicle control. After 7 days of implantation, Matrigel plugs were removed and fixed with 4% paraformaldehyde, then photographed with a digital camera. After Matrigel plugs were embedded and fixed in paraffin, neovascularization was determined by CD31 staining.

### Western Blot

The effect of RBF on VEGF-dependent angiogenesis signaling pathways was determined by Western blot assay. HUVECs (1 × 10^5^ cells/well) were seeded in a 6-well plate and incubated overnight in an incubator (37°C with 5% CO_2_). When the cell density reached about 80%, the cells were starved in a serum-free medium for 6 h. The serum-free medium containing different concentrations of RBF was then changed and continued to culture for 30 min and stimulated for 4 min with 100 ng/ml VEGF, subsequently. RIPA Lysis Buffer (Beyotime, Shanghai, China) supplemented with complete protease inhibitor cocktail and PhosSTOP phosphatase inhibitor cocktail (Roche, Rotkreuz, Switzerland) were used for cell lysis extraction. The concentration of protein was determined by BCA Protein Assay Kit (Beyotime, Shanghai, China) and equalized before loading. Then, 20 μg of membrane protein from each sample was applied to 7.5% SDS–PAGE. Polyvinylidene fluoride (PVDF) was incubated with primary antibody (Cell Signaling Technology, Danvers, MA) at 4°C and then co-incubated with horseradish peroxidase–coupled second antibody. The luminescent images were detected using ECL kits.

### Anticancer Therapy of Resibufogenin *In Vivo*

Mouse triple-negative breast cancer cells 4T1 (1 × 10^7^ cells/ml) and human triple-negative breast cancer cells MDA-MB-231 (5 × 10^7^ cells/mL) were suspended in PBS, and then inoculated in 6-week-old female BALB/c mice and female BALB/c nude mice on the fourth pair of fat pads (100 μl/mouse) to establish orthotopic model of breast cancer. Once the tumor volume reached ∼50 mm^3^, the all of mice were randomly divided into two groups (*n* = 6 per group): control group and RBF treatment group (10 mg/kg/day). The mice of the control group received intraperitoneal injection with oil and the mice in the RBF treatment group were injected with oil-containing RBF (10 mg/kg/day). The body weight, tumor length, and width of mice were monitored every 2 days. The formula for calculating tumor volume is as follows: tumor volume (mm^3^) = (length) × (width)^2^/2. After administration for 12 days, the mice were sacrificed and the tumor was dissected. And all of the tumors were photographed and weighed, and then fixed with 4% paraformaldehyde to prepare for paraffin section and immunohistochemical assay. The hematoxylin and eosin (H&E) staining evaluated tumor necrosis, and the immunohistochemical assay of CD31 staining was used to observe the tumor vessels according to the previous studies. All of the slices were photographed by photomicroscope. The tumor necrosis area, microvessel density, Ki67-positive cells, and TUNEL-positive cells of the slices were analyzed by Image-Pro Plus 6.0 software.

### Molecular Docking

Through the computer virtual docking of molecular operating environment (MOE), the molecular interaction between VEGFR2 and RBF was explored. First, the three-dimensional structure of RBF is generated by energy minimization in MOE. Then the x-ray crystals of VEGFR2 kinase domain and its ligands were obtained from the Protein Data Bank (http://www.rcsb.org). Two crystal structures of 3B8R and 3B8Q, which belong to DFG-in and DFG-out conformations, were selected to dock with RBF. The interaction between the molecules was analyzed and visualized by ligand interaction module and PyMOL.

### Statistical Analysis

All data were presented as mean ± SD. Statistical analysis and graphical representation of the data were performed using GraphPad Prism 6.0 (GraphPad Software, San Diego, CA). The differences between groups were examined with Student's *t*-test or ANOVA with Bonferroni's multiple comparisons tests. Differences were considered significant if the *p* value was less than 0.05.

## Results

### Resibufogenin Inhibits Viability of Human Umbilical Vein Endothelial Cells at Concentrations Not Affecting Triple-Negative Breast Cancers

As shown in Figures 1B,C, RBF inhibited the cell viability of HUVECs in a dose-dependent manner with a low half-maximal inhibitory concentration (IC_50_) value, 3 μM. The cell viability of HUVECs was distinctly inhibited at RBF concentration of 10 μM. In contrast, RBF had almost no obvious effect on the viability of the TNBCs 4T1 and MDA-MB-231 at the same concentrations of HUVECs. These results proved that ECs are more sensitive to RBF than 4T1 and MDA-MB-231 cells. Moreover, RBF did not exert significant anti-proliferative effect toward HUVECs at the dose from 0.3 to 30 μM within 12 h.

### Resibufogenin Inhibits Endothelial Cell Migration, Invasion, and Tube Formation

**FIGURE 2:**
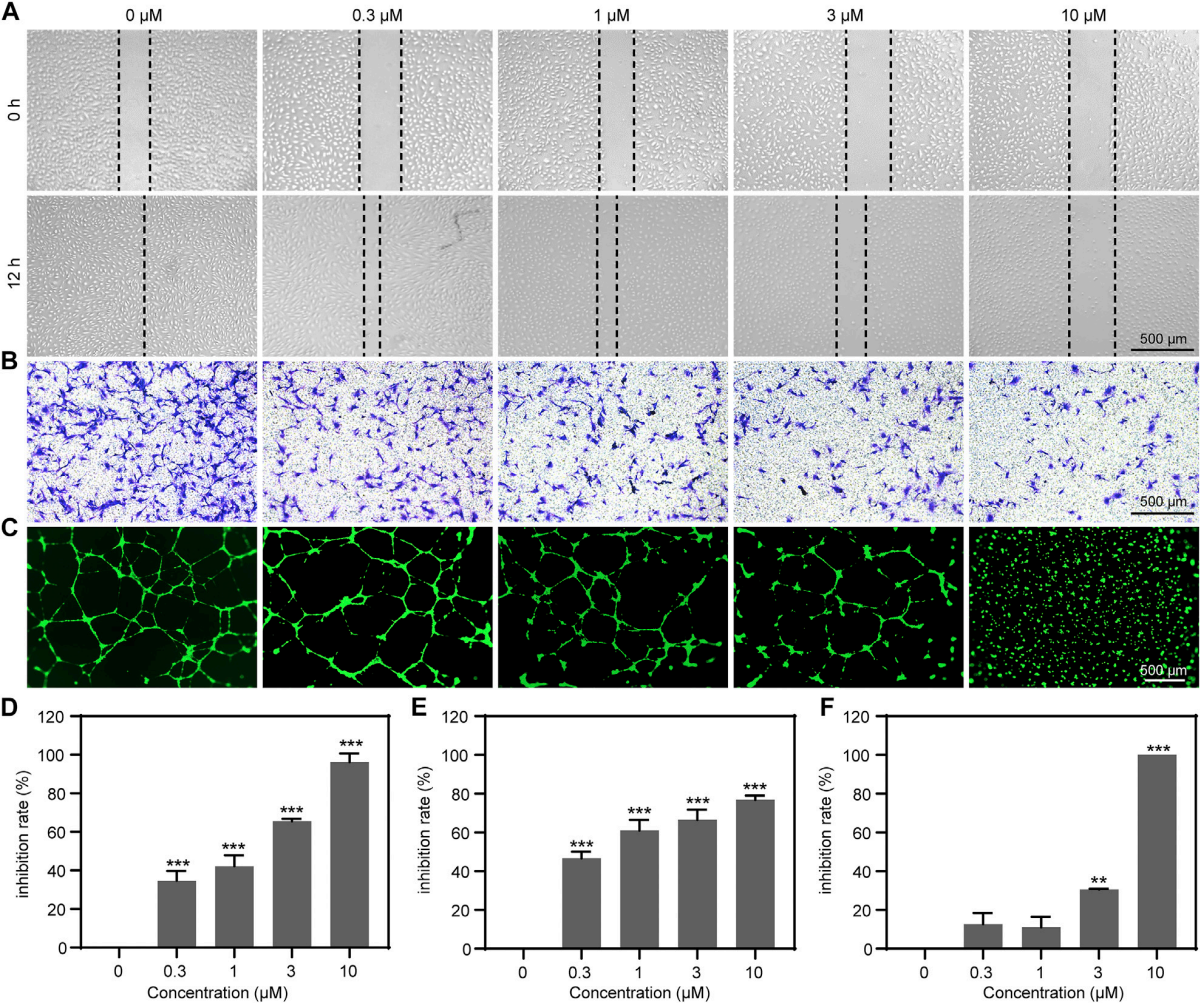
RBF significantly suppressed HUVEC motility, migration, and tube formation. **(A)** RBF inhibited HUVEC migration in wound healing assay. Dotted lines indicated the boundary of initial scraping. **(B)** RBF suppresses HUVEC vertical migration in transwell assay. HUVEC migration after 8 h treatment of RBF was assayed using transwell Boyden chamber. **(C)** RBF inhibited tube formation of HUVECs. HUVECs were placed in the Matrigel coated 96-well plate. After 10 h, the tubular structures were photographed. **(D)** The inhibition rate of wound healing after treatment with different concentrations of RBF. **(E)** The inhibition rate of the migrated cells after treatment with different concentrations of RBF. **(F)** The inhibition rate of tube formation after treatment with different concentrations of RBF. Values are expressed as mean ± SD, *n* = 4. **p < 0.01 and ***p < 0.001 as compared with control.

The migration of ECs is the important step in the process of angiogenesis (Varinska et al., 2018). Thus, we performed a wound healing assay and transwell migration assay to investigate the effect of RBF on the horizontal and vertical migration ability of HUVECs. As shown in Figures 2A,D, RBF dose-dependently inhibited the lateral migration of ECs, and it was obvious that the migration ability of HUVECs was completely inhibited at the concentration of 10 μM. The transwell assay (Figures 2B,E) showed that RBF could inhibit the vertical migration of HUVECs to the bottom chamber at 0.3 μM. To evaluate the antiangiogenesis ability of RBF, we performed a tube formation assay to verify the effects of RBF on tube formation of HUVECs on a Matrigel substratum. HUVECs could form a complete tubular network after VEGF stimulation (Figures 2C,F), while RBF markedly restrained HUVECs tube formation at the concentration of 3 μM. These results suggested that RBF has a strong inhibitory effect on HUVEC motility, migration, and tube formation at the nontoxic concentrations.

### Resibufogenin Inhibits Human Umbilical Vein Endothelial Cells Spheroid Sprouting

**FIGURE 3:**
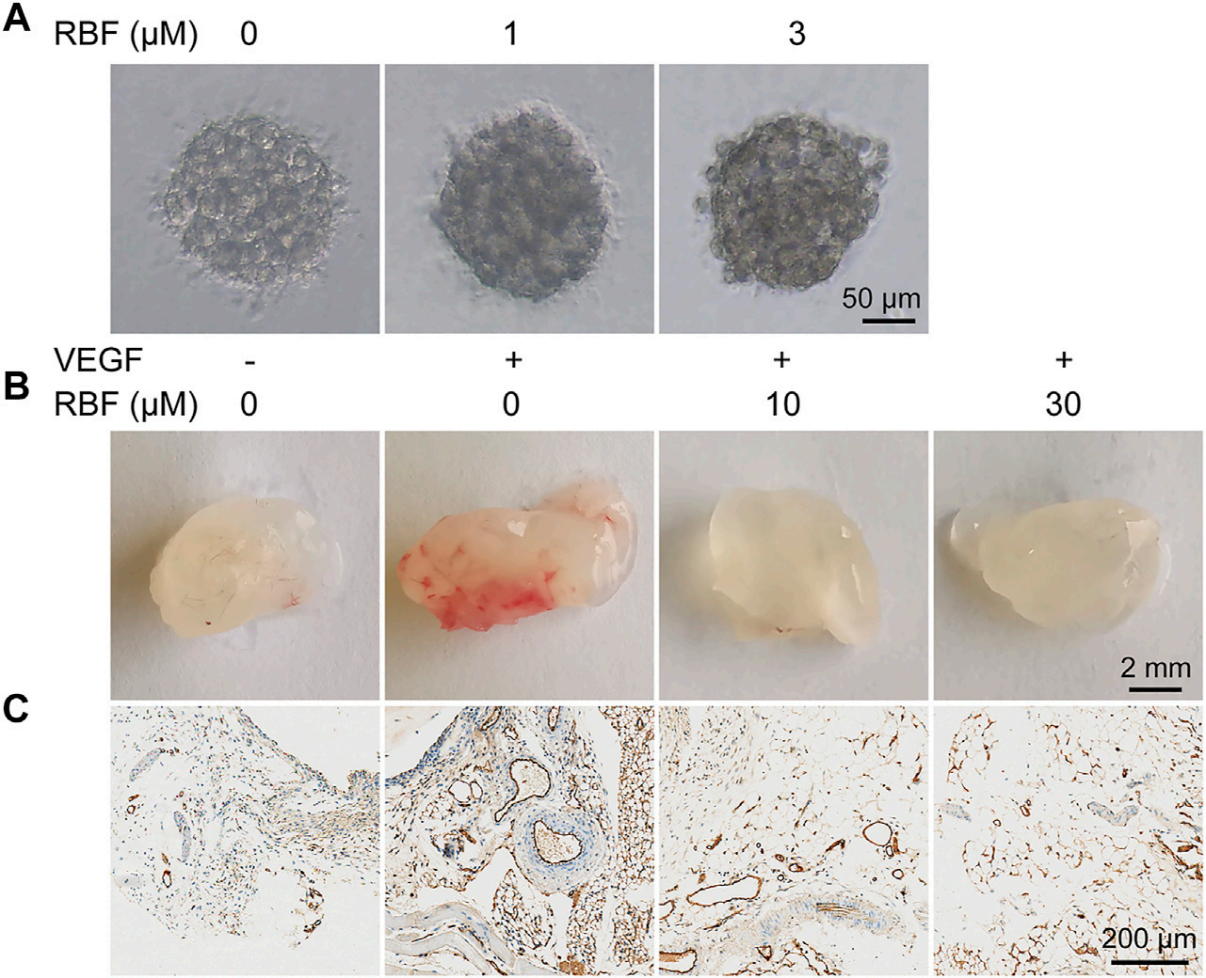
RBF blocked angiogenesis in 3D HUVEC spheroid and Matrigel plug. **(A)** Sprouting from HUVEC spheroids stimulated with VEGF were inhibited by RBF. **(B)** Representative photographs of Matrigel plug treated with different concentrations of RBF. **(C)** Representative CD31 staining sections showing the vessel in each Matrigel plug.

In the sprout formation assay, HUVECs were formed a single spheroid and then embedded in a 3D collagen matrix. In the control group, the HUVEC spheroid was sprouting significantly under the stimulation of VEGF_165_. However, when treated with different concentrations of RBF, the sprouts became thinner. Sprouting was almost completely inhibited by the treatment with RBF at 3 µM (Figure 3A). These results suggested that RBF has an inhibitory effect on HUVEC spheroid sprouting.

### Resibufogenin Inhibits Angiogenesis in Matrigel Plugs *In Vivo*

Next, we performed the Matrigel plug assay to further explore whether RBF could inhibit angiogenesis *in vivo*. As illustrated in Figure 3B, compared with the PBS group, Matrigel plugs mixed with VEGF exhibited obvious red area after 1 week of implantation, indicating a large number of new blood vessels in Matrigel plugs. In contrast, Matrigel plugs mixed with RBF were almost colorless and transparent, suggesting almost no angiogenesis existed. These results indicated that RBF could significantly inhibit neovascularization in the Matrigel plugs *in vivo*. The existence of blood vessels was verified by staining of CD31, a specific marker on the surface of ECs (Privratsky and Newman, 2014; Lertkiatmongkol et al., 2016). The results in Figure 3C displayed that RBF (10 μM) had a significant inhibitory effect on angiogenesis stimulated by VEGF. The number of blood vessels in the high-dose group (30 μM) was similar to that in the PBS alone group.

### Resibufogenin Suppressed the Activation of VEGFR2-Mediated Signaling Pathway

**FIGURE 4:**
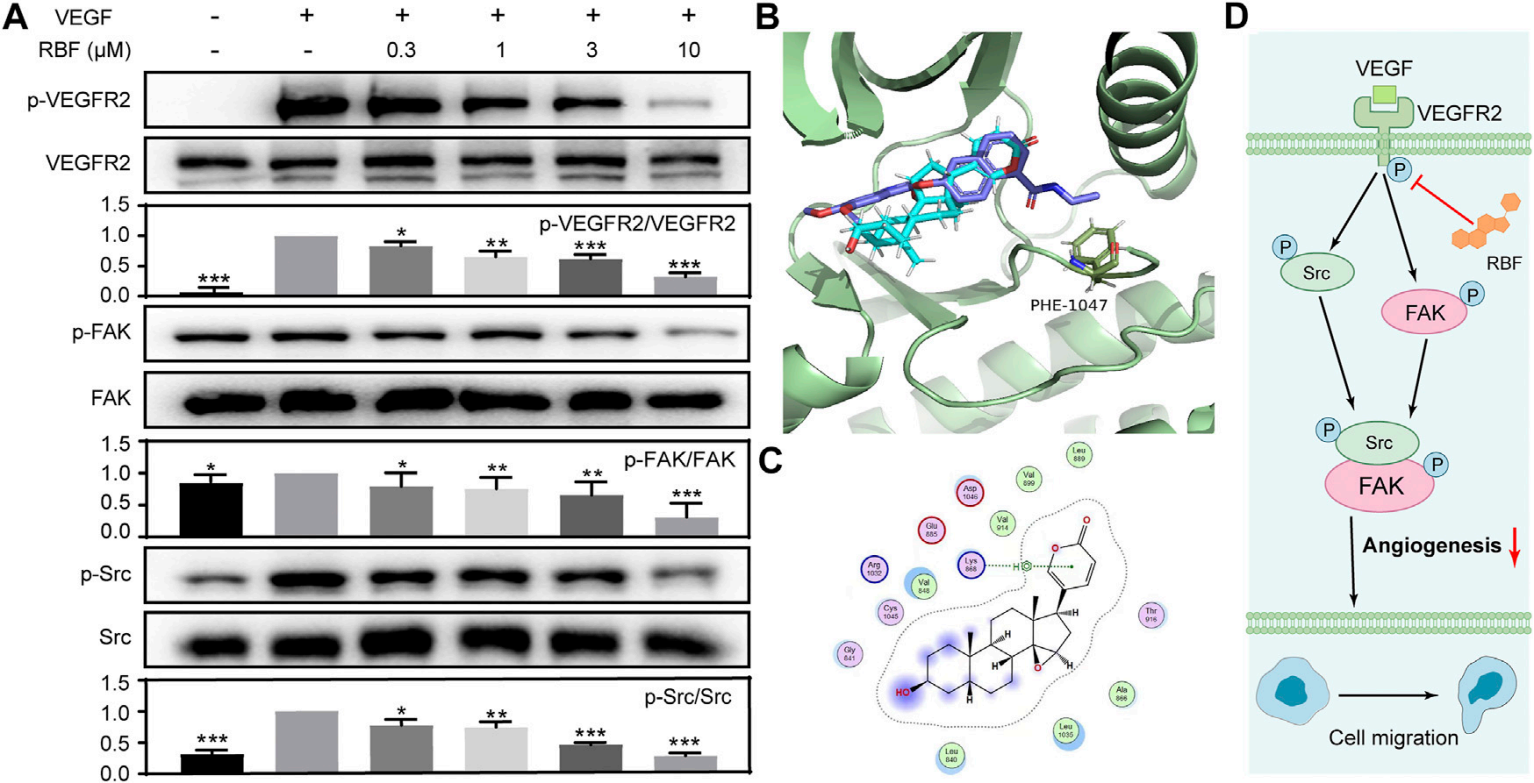
RBF inhibited the phosphorylation of VEGFR2 and its downstream signaling molecules in endothelial cells. **(A)** RBF inhibited the activation of VEGFR2 and its downstream signaling kinases in HUVECs. HUVECs were pretreated with various concentrations of RBF for 0.5 h followed by the stimulation with 100 ng/ml of VEGF for 4 min. The activation of VEGFR2 and its downstream cascade (FAK and Src) were analyzed by Western blot. The quantified results were shown as the grayscale ratio of phosphorylated protein to the total protein. *p < 0.05, **p < 0.01, and ***p < 0.001 as compared with control (HUVECs stimulated by VEGF), *n* = 3. **(B)** The predicted binding interactions between RBF and VEGFR2. Side chains of crucial residues in the binding site are shown as blue sticks. **(C)** Two-dimensional interaction map of RBF and ATP binding pocket of VEGFR2 kinase domain was visualized by ligand interaction module. **(D)** Signaling pathway diagram of RBF-mediated antiangiogenesis.

The VEGF signaling pathway and its main receptor, VEGFR2, could stimulate tumor angiogenesis in most solid tumors (Simons et al., 2016). To verify the mechanisms involved in the antiangiogenic function of RBF, we first examined whether RBF could affect VEGF-mediated phosphorylation of VEGFR2 by Western-blotting assay. It was shown that the expression of tyrosine phosphorylation of VEGFR2 was significantly increased after VEGF stimulation, and RBF could inhibit the phosphorylation level of VEGFR2 in a dose-dependent manner (Figure 4A). As known, the downstream signaling pathway could be activated by the VEGFR2, which regulate multiple activities of ECs, including migration, proliferation, and survival (Simons et al., 2016; Wang et al., 2020). We further investigated the effect of RBF on the expression level of downstream proteins of VEGFR2. The results suggested that RBF could downregulate the level of phospho-FAK and phospho-Src. Taken together, the above results proved that RBF could inhibit the phosphorylation of VEGFR2, FAK, and Src to block the angiogenesis ability of ECs.

### Resibufogenin Competitively Bound ATP-Binding VEGFR2 Kinase Domain

As RBF downregulate the phosphorylation of VEGFR2 and its downstream signal molecules, it was speculated that RBF may be an inhibitor of VEGFR2 kinase. So, molecular docking simulation was carried out to predict the possible binding mode between RBF and VEGFR2 kinase domain. Studies have shown that VEGFR2 inhibitors can be divided into two types according to different binding modes, namely, type I (conformational complex with DFG-in) and type II (conformational complex with DFG-out) protein kinase inhibitors (Huang et al., 2012). Type I inhibitors affect phosphorylation of VEGFR2 by competitively binding ATP- binding VEGFR2 kinase domain, thereby preventing ATP from providing phosphate groups. While type II inhibitors inhitbit phosphorylation by occupying the space position of Phe1047 in active conformation (DFG-in) and preventing VEGFR2 from transforming from inactive to active (Weiss et al., 2008; Huang et al., 2012). Then, DFG-in and DFG-out protein states were selected for docking. As shown in Figures 4B,C, RBF could bind to the DFG-in protein state and cannot penetrate into the Phe1047 pocket to further prevent VEGFR2 activation. RBF mainly interacted with amino acid residues, including Leu840, Val848, Ala866, Leu889, Val899, Val914, and Leu1035 via the hydrophobic interaction. There was also H–pi interaction between the arene moiety of RBF and the key residues of Lys868. These results indicated that RBF was a Type I inhibitor which inhibited the phosphorylation of VEGFR2.

### Resibufogenin Inhibited Tumor Angiogenesis and Growth in 4T1 and MDA-MB-231 Orthotopic Mouse Models

**FIGURE 5:**
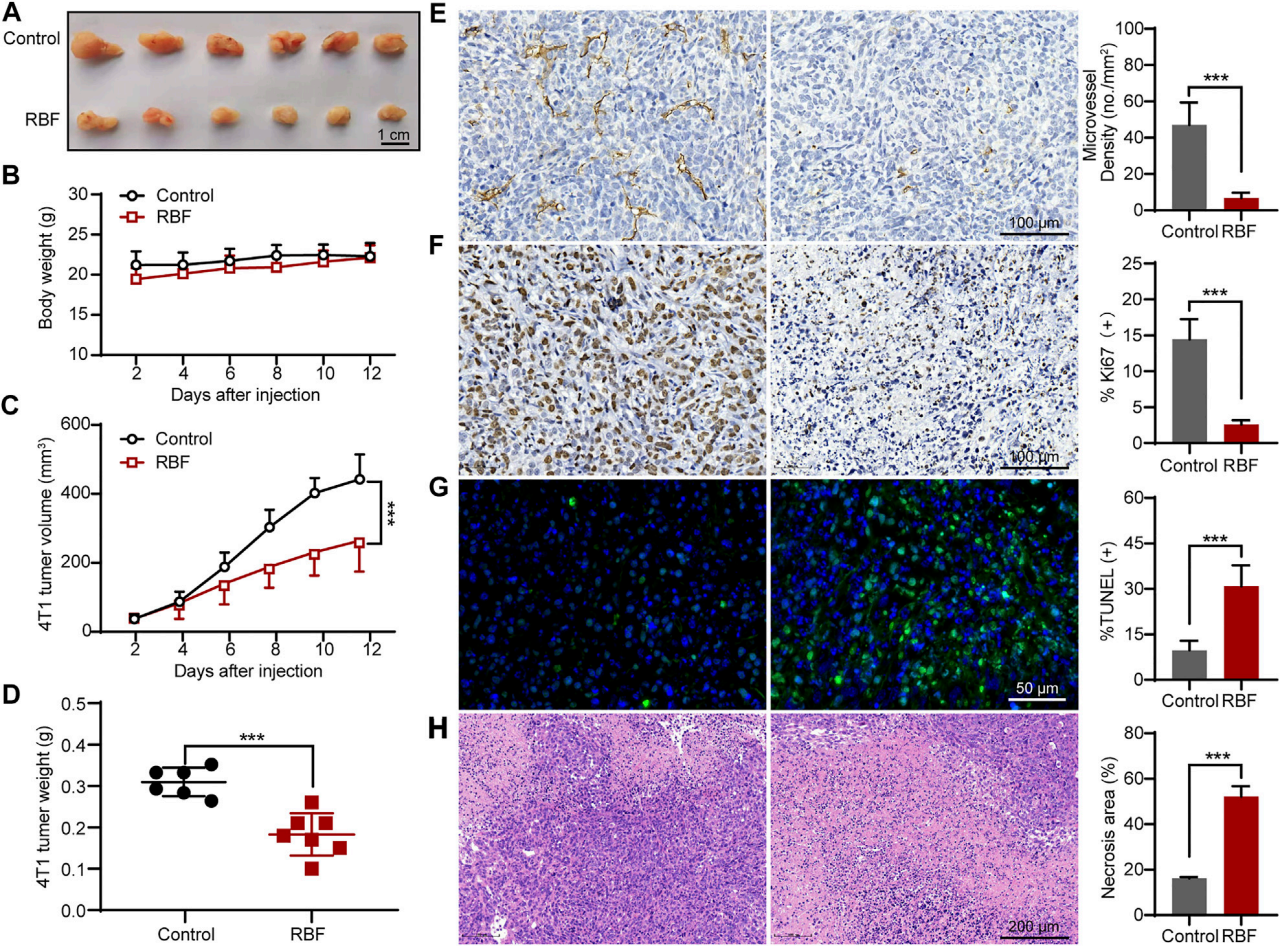
RBF inhibited tumor angiogenesis and growth in 4T1 xenograft mice model. **(A)** Photograph of the resected tumors from the control and RBF groups. **(B)** Mice body weight. **(C)** RBF treatment decreased the tumor weight compared with the control. **(D)** RBF treatment delayed the tumor growth compared with the control. **(E)** Intratumoral CD31-positive vessel (microvessel density, MVD). **(F)** Ki67-positive proliferation tumor cells. **(G)** TUNEL-positive apoptotic tumor cells **(H)** H&E staining sections and statistical analysis of necrosis area. All values were shown as mean ± SD, *n* = 5. **p < 0.01 and ***p < 0.001 as compared with control.

In order to investigate the inhibiting effects of RBF on tumor angiogenesis and growth *in vivo*, the tumor models were first established by *in situ* inoculation of 4T1 cells in BALB/c mice (Duan et al., 2019; Tsui et al., 2019). RBF was injected intraperitoneally at a dose of 10 mg/kg/day for 12 days. The results showed (Figures 5A,C,D) that RBF significantly inhibited the growth of tumor, the tumor volume was 246.15 ± 69.9 mm^3^, and the average tumor weight was only 0.17 ± 0.04 g, whereas the tumor volume was 471.89 ± 45.1 mm^3^ and the average tumor weight was 0.31 ± 0.03 g in the control group. In addition, there was no significant change in the body weight of mice at this dose (Figure 5B), indicating that RBF had no obvious toxicity to the mice at the curative dose. Immunohistochemistry and pathological examination showed that RBF not only effectively inhibited tumor cell proliferation and increase tumor necrotic area but also reduced tumor microvessel density and elevate TUNEL-positive cells.

**FIGURE 6:**
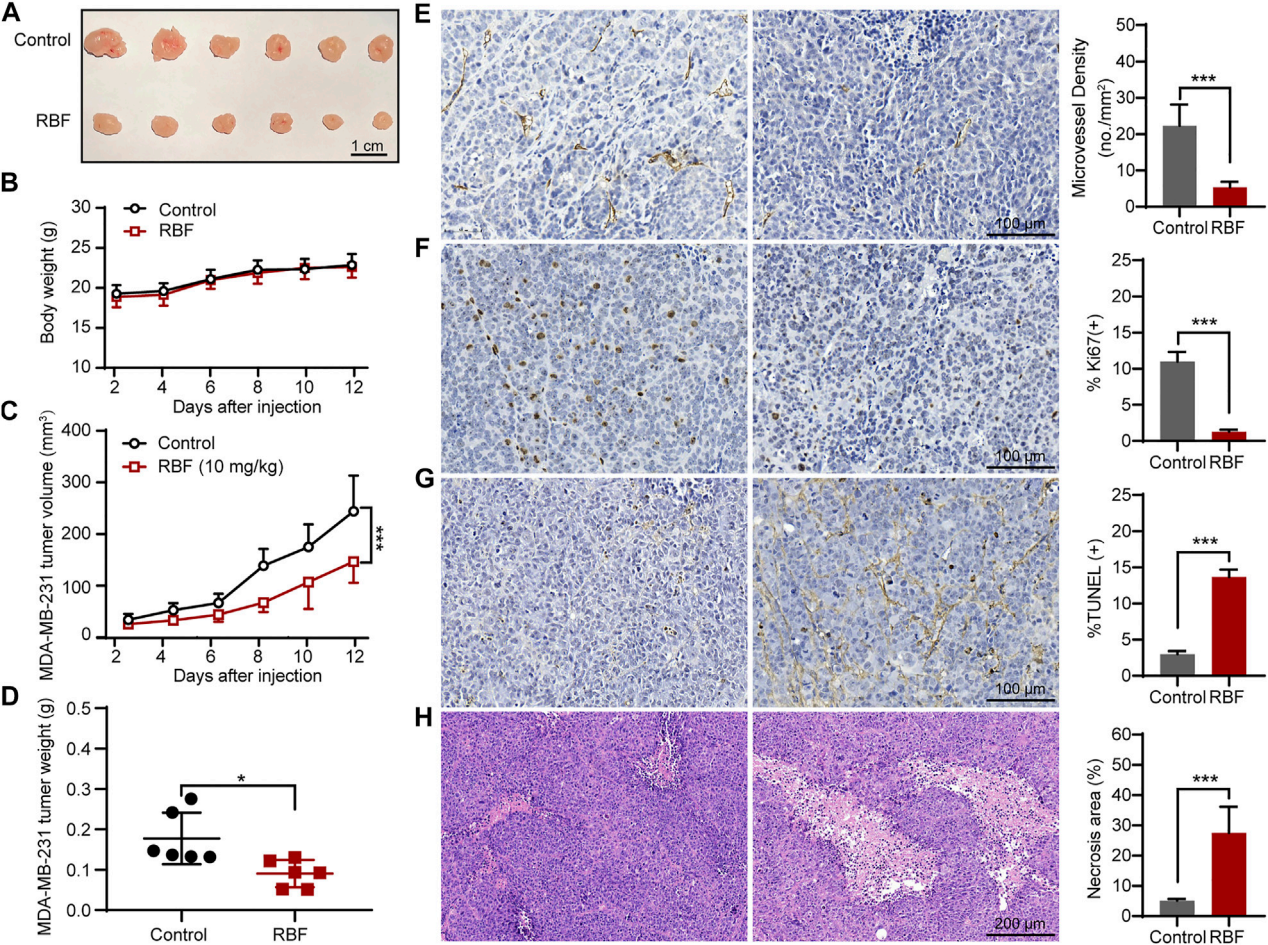
RBF inhibited tumor angiogenesis and growth in MDA-MB-231 xenograft mice model. **(A)** The photograph of the resected tumors from the control and RBF groups. **(B)** Mice body weight. **(C)** RBF treatment decreased the tumor weight compared with the control. **(D)** RBF treatment delayed the tumor growth compared with the control. **(E)** Intratumoral CD31-positive vessel (microvessel density, MVD). **(F)** Ki67-positive proliferation of tumor cells. **(G)** TUNEL-positive apoptotic tumor cells **(H)** H&E staining sections and statistical analysis of the necrosis area. All values were shown as mean ± SD, *n* = 5. **p < 0.01 and ***p < 0.001 as compared with control.

Meanwhile, we also established another TNBC model by *in situ* inoculation of human TNBC cell line (MDA-MB-231) into BALB/c nude mice (Shi et al., 2019; Kachamakova-Trojanowska et al., 2020; Xu et al., 2020). As shown in Figure 6C, RBF also showed significant antitumor activity in this tumor model, and the tumor volume was 146.77 ± 37.5 mm^3^ much smaller than that in the control group (244.31 ± 62.9 mm^3^). In contrast to untreated controls, RBF-treated group showed a profound decrease in the number of CD31-positive microvessel and Ki67-positive cells, while the rate of TUNEL-positive cells and the area of tumor necrosis increased. These results suggested that the antiangiogenic activity of RBF effectively contributed to its antitumor effect *in vivo*.

## Discussion

Neovascularization is the critical characteristic of solid tumors to contribute tumor rapid progression and metastasis. Therefore, targeting tumor blood vessels has been considered as a reasonable approach to the treatment of various malignancies (Rajabi and Mousa, 2017). Bevacizumab, a recombinant humanized monoclonal IgG1 antibody that binds to VEGF, provides a new hope of improved survival for patients with intractable TNBC in combination with paclitaxel and capecitabine (Liu et al., 2020). However, this neoadjuvant therapy cannot fully meet the expectations of patients for higher overall survival owing to acquired resistance. Given that, it is imperative to explore alternative novel antiangiogenic agents to improve the therapeutic effectiveness.

Active small components derived from TCM have been demonstrated to possess excellent bioactivity with low toxicity in the treatment of many diseases. Taking these advantages, we successfully identified a TCM *Bufo bufonis*–derived small molecule, RBF, which have high selectivity between TNBCs and HUVECs. Multiple mechanisms for RBF antitumor activity have been elucidated, but none of them touched its antiangiogenic activity in breast cancers, especially TNBC. We first demonstrated the potent antiangiogenic ability and mechanisms of RBF *in vitro* as well as the anti-TNBC effect *in vivo*.

We successfully proved that RBF could perform the antiangiogenic function toward migration, invasion, and tube formation of HUVECs in a dose-dependent inhibition. Meanwhile, we found that the sprouting 3D spheroid sprouts were thinner and shorter after treatment of RBF. The Matrigel plug assay was used to verify the antiangiogenesis effect of RBF *in vivo*. Then, we constructed 4T1 and MDA-MB-231 orthotopic mouse models to evaluate the therapeutic effect of RBF through antiangiogenic potency. As expected, the results exhibited that RBF can not only suppress the growth of mouse TNBC *in vivo*, but also inhibit human TNBC progression in mice through the successful blocking effect on tumor-related angiogenesis, thereby highlighting the potential clinical transformation of RBF. Immunohistochemical assay further revealed that RBF could significantly increase the necrotic area of tumor, inhibited tumor cell proliferation, and promoted apoptosis. More importantly, the intratumoral CD31-positive vessel in the RBF treatment group decreased pronouncedly, suggesting that its antitumor effect was closely related to antiangiogenesis. Collectively, these *in vitro* and *in vivo* results both suggested that the antiangiogenic activity of RBF played a critical role in suppressing tumor growth *in vivo* (Guo et al., 2020).

Among angiogenic factors, VEGF has the strongest effect to the process of angiogenesis. VEGFR2 plays a principal role in mediating VEGF-induced series of downstream signals of angiogenesis (such as Akt pathway, NF- κB pathway, and MAPK pathway) that subsequently promote the activation of endothelial cells (Simons et al., 2016). Thus, targeting VEGFR2 signaling pathway to inhibit tumor angiogenesis is regarded as vital strategy. We also observed that RBF dose-dependently decreased the VEGF-induced VEGFR2 phosphorylation and its downstream signals, including FAK and Src. Molecular docking test indicated that RBF could locate at the ATP-bound VEGFR2 kinase domain through hydrophobic interaction and H–pi interaction, thereby blocking the phosphorylation of VEGFR2. Such bioinformatics of the binding pattern of RBF and VEGFR2 can help us better understand the antiangiogenic effect of RBF, and we could reinforce this binding by chemical structure modification of RBF.

In conclusion, we successfully elucidated the antiangiogenic effect and corresponding mechanism of RBF on the HUVECs by attenuating VEGFR2 signal pathway. More importantly, this new antitumor mechanism further contributed to slow tumor growth and lower microvessel density in two TNBC mice, which provide a promising candidate for angiogenesis in TNBC treatment.

## Data Availability

The original contributions presented in the study are included in the article/Supplementary Material, further inquiries can be directed to the corresponding authors.
